# Phytochemical Characterization of Purple Coneflower Roots (*Echinacea purpurea* (L.) Moench.) and Their Extracts

**DOI:** 10.3390/molecules28093956

**Published:** 2023-05-08

**Authors:** Ani Petrova, Manol Ognyanov, Nadezhda Petkova, Petko Denev

**Affiliations:** 1 Laboratory of Biologically Active Substances-Plovdiv, Institute of Organic Chemistry with Centre of Phytochemistry, Bulgarian Academy of Sciences, 139 Ruski Blvd., 4000 Plovdiv, Bulgaria; ani.petrova@orgchm.bas.bg; 2 Department of Organic Chemistry and Inorganic Chemistry, Technological Faculty, University of Food Technologies, 26 Maritza Blvd., 4002 Plovdiv, Bulgaria; petkovanadejda@abv.bg

**Keywords:** purple coneflower (*Echinacea purpurea*) roots, glycerol extraction, cichoric acid, inulin, polysaccharides, characterization, UHPLC, FTIR

## Abstract

*Echinacea purpurea* is a perennial plant that belongs to the Asteraceae family. It has a wide range of applications mainly in the treatment and prevention of inflammations in the respiratory system. The current study aimed to perform a phytochemical characterization of purple coneflower (*Echinacea purpurea*) roots and their extracts (water, 40%, 50%, 60% ethanol, and 60% glycerol). Phytochemical characterization was carried out by gravimetric, spectrophotometric, and chromatographic methods. *Echinacea* roots were characterized by a low lipid (0.8%) content. In contrast, carbohydrates (45%) and proteins (20%) occupied a large part of the dry matter. Amongst the extracts, the highest yield was obtained using water as a solvent (53%). Water extract was rich in protein and carbohydrates as fructans (inulin) were the most abundant carbohydrate constituent. The most exhaustive recovery of the phenolic components was conducted by extraction with 40% ethanol and 60% glycerol. It was found that water is the most suitable extractant for obtaining a polysaccharide-containing complex (PSC) (8.87%). PSC was composed mainly of fructans (inulin) and proteins with different molecular weight distributions. The yield of PSC decreased with an increasing ethanol concentration (40% > 50% > 60%) but the lowest yield was obtained from 60% glycerol extract. The obtained results showed that *Echinacea* roots contained a large amount of biologically active substances—phenolic components and polysaccharides and that glycerol was equally efficient to ethanol in extracting caffeic acid derivatives from purple coneflower roots. The data can be used for the preparation of extracts having different compositions and thus easily be incorporated into commercial products.

## 1. Introduction

Purple coneflower or echinacea (*Echinacea purpurea* (L.) Moench.) is an herbaceous perennial plant, belonging to the Asteraceae (Compositae) family. The plant is one of the most widely cultivated herbs due to its valuable health-promoting properties and ornamental purposes. In traditional folk medicine, the herb is mainly used for chemotherapy of infectious diseases of the upper respiratory system, including influenza and wound healing [1]. In general, echinacea is applied in the form of an extract prepared by a decoction or maceration of the dried, whole, or cut aerial or underground parts of the plant. 

Different biologically active constituents such as alkamides, caffeic acid derivatives (cichoric acid), essential oils, and polysaccharides are believed to be involved in the immunomodulatory properties of echinacea [1,2]. However, scientists have not reached a consensus on the primary active component. On the contrary, they have supposed that a synergistic effect existed between the different constituents in the preparations [3]. Nevertheless, European pharmacopoeia clearly defines caftaric acid and cichoric acid as markers for the evaluation of coneflower herbal material [4]. For instance, the flowering aerial parts should contain a minimum of 0.1% of both components, whereas their amount in the roots should not be lower than 0.5%. Unfortunately, many echinacea-containing products do not have any quantitative data on the above-mentioned phenolic constituents. What is more, the standardization of extracts by only one component would lead to the manufacturer’s desire to provide enough quantity of this compound. However, the coneflower root possesses a cocktail of compounds very different in nature [5,6]. In addition, the extracts’ composition strongly depends on the extraction condition used such as solvent, time, temperature, plant/solvent ratio, quality of raw material, etc. Thus, extracts with varying and unknown compositions can be prepared in which one of the active constituents is missing or present in very small quantities. In general, 70% ethanol in water is used for the extraction of caffeic acid derivatives from Echinacea roots [4]. However, this solvent is not suitable for polysaccharide extraction, which is readily extractable with pure water. Another underestimated candidate for solvent is glycerol. It is an inexpensive and nontoxic natural substance that is a principal byproduct of the biodiesel industry resulting from the transesterification process. It is considered a renewable feedstock for the production of various chemicals [7]. Moreover, water-glycerol mixtures are suitable extractants for phenolic compounds from olive leaves [8], which triggered many subsequent studies on the effectiveness of this solvent on different plant materials. 

Despite the widespread use of Echinacea in herbal medicines and food supplements, more detailed information is not yet available on the rate of extraction of important components such as polysaccharides and major phenolic constituents from the roots with different solvents. To our knowledge, there are no studies in the literature conceived to investigate the effectiveness of different extractants (water, ethanol, and glycerol) for the concomitant extraction of polyphenols and polysaccharides from purple coneflower roots and moreover, glycerol effectiveness for the extraction of *E. purpurea* root polysaccharides have not been investigated. Therefore, the current study mainly investigates the composition of purple coneflower root and the major phytochemical constituents of different extracts obtained by water, water-ethanol, and water-glycerol mixtures. The result of the study could find practical application in the development and formulation of ethanol-free *E. purpurea* food supplements and nutraceuticals. 

## 2. Results

### 2.1. Characterization of Initial Plant Material

Initially, we conducted a composition analysis of the plant material before the examination of the extracts. The results are summarized in Table 1. It can be seen that carbohydrates were the major fraction (45% *w*/*w*) of purple coneflower roots’ dry matter. Compositional data revealed that polysaccharide constituents (~63%) predominated in comparison with soluble sugars (2%) which occupied not more than 5% of total carbohydrates. Fructose was the major soluble sugar, followed by glucose and sucrose. As shown (Table 1), cellulose, a component of the primary cell walls, was observed to be the most represented polysaccharide constituent (32%) in the root, while uronic acids constituting an acidic polysaccharide such as pectin, were the second most abundant component (17%). Echinacea roots were characterized by a high total fructan content (16%). Inulin was the most abundant fructan, accounting for more than 88% of the total amounts of total fructans detected. Inulin-type fructooligosaccharides 1-kestose and nystose were found in lower amounts (<10%).

Furthermore, plant material was characterized by a high amount of crude protein (20.2%). It should not be excluded that this value was overestimated due to the presence of nonprotein nitrogen compounds (alkamides, etc.) in the root, which was previously indicated [6]. Other plant constituents such as moisture and crude lipids were found in small quantities. Ash content (4.7%) did not exceed that specified in European pharmacopoeia (≤9.0%) [4]. 

The quantitative data from the liquid-chromatography assay of phenolic acids (Figure S1) are also included in Table 1. Cichoric acid represented the major derivative of caffeic acid, and together with caftaric acid, they occupied nearly 60% of total phenolic compounds. The sum of both acids was 0.84%, suggesting that the used plant material met the quantitative requirement of the European pharmacopoeia (≥0.5%) [4]. Tannins and flavonoids occupied a small part of the total phenolic content.

### 2.2. Characterization of Root Extracts

After the primary characterization of the coneflower root, we decided to concentrate our efforts on the preparation of extracts with different solvents (water, 40%, 50%, 60% ethanol, and 60% glycerol) widely used in the food and pharmaceutical industries. The results of the different analyses are included in Table 2. From the table, it is evident that the water extract yielded the highest amount (53%), whereas the yield of ethanolic extracts decreased with increasing the ethanol concentration in the solvent: Y_E40%_ > Y_E50%_ > Y_E60%_. Not surprisingly, many cell-wall components, especially polymers, were insoluble in a higher concentration of organic solvents by comparison with water. Unfortunately, we were not able to determine the yield of glycerolic extracts due to the impossibility of removing the solvent from the extract and residue. 

Impressively, the extracts consisted mainly of protein (26–29%), which was accompanied by lower levels of carbohydrates (8–11%). Amongst extracts, E_H2O_ was characterized by a higher protein content in comparison with ethanolic extracts. It seems that water extraction solubilized 76% of the total protein content, present in the initial roots. By contrast, the ethanolic solvents recovered lower amounts of protein 50%, 44%, and 42% with increasing the ethanol concentration in the solvent. There was a tendency to reduce the extraction of total carbohydrates when extracting roots with a highly concentrated organic solvent (13% > 9% > 7% > 6%). 

With increasing the ethanol concentration in the solvent, the total fructan solubility was reduced from 40% to 24%. It can be seen that water solubilized more completely inulin (59%), while 40% ethanolic solvent extracted not more than 46% from the initial inulin. Therefore, the extraction residue still contained more than half of the inulin present in the roots (56%). On the other hand, fructooligosaccharides were solubilized more fully by water because 80% of them were recovered in the extracts (Table 2). Surprisingly, uronic acids, which constitute acidic polysaccharides and decorate some arabinogalactan-protein polymers, were hardly solubilized by water and, probably, harsher extraction conditions should be selected (high temperature, etc.). As regards phenolic constituents, water extract was characterized by low phenolic content (1.16%). By contrast, ethanolic extracts consisted of a higher amount of phenolic compounds (1.46–1.53%). Interestingly, phenolic constituents were more soluble in 60% glycerol (1.70%), than in water and water-ethanol solutions. It seems that cichoric acid and caftaric acid were poorly soluble in water, but their extractability increased from 8% (water) to 32% employing 60% ethanol. A 60% aqueous solution of glycerol was an equally efficient extractant of caffeic acid derivatives like 60% ethanol. 

The results of our experiment indicated that water was a better solvent for some carbohydrate constituents (especially fructans) and proteins, but not suitable for phenolic components. Thus, 40% ethanol could serve as a ‘compromise’ solvent able to solubilize equally well different echinacea-root components.

### 2.3. Characterization of Extracts Prepared from the Pre-Extracted Root 

As it was mentioned above in the Results Section 2.2, most cell-wall constituents were insoluble in a higher concentration of organic solvents, and thus their recovery was very low in comparison with water. To examine the potential of residual 40%, 50%, and 60% ethanol-extracted root material for recycling and additional utilization, they were re-extracted with water and 60% glycerol. Thus, efficient utilization of these byproducts would lead to additional and increasing costs for the extracts. Table 3 summarises the results of the analyses.

It can be seen that the yield of water extracts increased with increasing the ethanolic concentration of solvent used for primary extraction: Y_E40%/H2O_ < Y_E50%/H2O_ < Y_E60%/H2O_. The yield of E_60%/H2O_ was two-times higher than E_40%/H2O_. As shown (Table 3), crude protein was predominantly present in the water extracts, but those amounts represented not more than 15% of the total protein content of the roots. For example, it was easily calculated that after extracting the roots with 40% ethanol followed by additional water extraction of the residue, 65% of the total protein content was recovered. However, it was not difficult to calculate precisely that soluble sugars in E_40%/H2O_ comprised less than 1% of the total available carbohydrates in the roots. This example suggested that sugars have already been extracted by the first extractant (40% ethanol). An extra amount of fructans can be extracted by water (E_40%/H2O_ < E_50%/H2O_ < E_60%/H2O_) and at a lower extent by 60% glycerol. It seems that a part of 60% ethanol-unextracted fructans was additionally solubilized by water. A further inspection of the data in Table 3 suggested that two-step extraction with 60% ethanol and water was closely equivalent to the 50% ethanolic extraction concerning fructans amount. The total phenolics were found at lower levels (>3 times) in E_40%/H2O_, E_50%/H2O_, and E_60%/H2O_ in comparison with E_40%_, E_50%_, and E_60%_, suggesting that a larger part of them was extracted initially. Nevertheless, similar to fructans, the phenolic content of water/60% glyceric extracts increased with increasing the ethanolic concentration of the solvent used for primary extraction: E_40%/H2O_ < E_50%/H2O_ < E_60%/H2O_, indicating that some phenolic constituents were freely solubilized with water and glycerol. 

### 2.4. Characterization of Polysaccharide Constituents 

We were very interested in revealing more about the polysaccharide constituents of root extracts because they are the most important plant cell-wall components. It is considered that they are one of the main active components of purple coneflower roots responsible for biological activity [3,5,6] but, paradoxically, many qualitative and quantitative sets of data about different extracts have not yet been thoroughly collected. The quantitative data are included in Table 4. From the table, it is evident that water solubilized the highest quantity of polysaccharides (8.9%), followed by 40% and 50% ethanol (5.8% and 5.4%). Logically, an increase in the dehydrating power of the solvent resulted in the precipitation of the polymer in cell walls and thus obtained a lower yield (Y_E40%_ > Y_E50%_ > Y_E60%_). For example, the yields of PSC_60%_ and PSC_60%G_ were 2.5 and nearly 4 times lower than PSC_H2O_. The lowest yield of PSC was obtained by 60% glycerol. The increasing extractability of polysaccharides from the residue with water can be easily explained, keeping in mind that a large part of them was retained during the primary extraction. However, the solubility of polysaccharide constituents was still poor when using 60% glycerol for re-extraction.

Table 4 shows that polysaccharide constituents were accompanied by higher levels of protein (11–18%). Other minor polysaccharide components were uronic acids. This suggests that PSCs did not consist of pectin-type polysaccharides. On the contrary, the obtained PSCs were characterized as an inulin-type polysaccharide complex having 20–44% fructan content. It is interesting to note that fructans were the major constituents of PSCs comprising between 80% and 98% of the total carbohydrates (PSCs), whereas the other components made up not more than 2–15%. 

**Figure 1:**
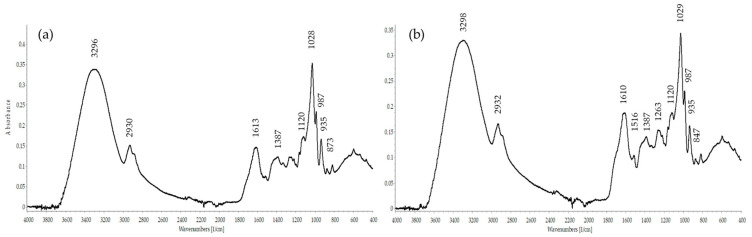
FT-IR spectra of the PSCs isolated from purple coneflower root extracts: (**a**) PSC_H2O_; (**b**) PSC_60%G_.

The inulin nature of polysaccharides was additionally confirmed by spectral analysis. The FT-IR spectra of the studied PSCs are presented in Figure 1. The FT-IR spectra of PSC_H2O_ and PSC_60%G_ contained typical bands for inulin-type fructans. A broad band at 3296–3298 cm^−1^ was assigned to O–H stretching vibrations and it was connected with inter- and intramolecular hydrogen bonds in the carbohydrate structure. The weak bands at 2930–2932 cm^−1^ were due to C–H asymmetric stretching vibrations, while bands at 2884 cm^−1^ were assigned with symmetric stretching vibrations of C–H. The bands at 1610 cm^−1^ were typical for the absorption of water. The bands at 1387 cm^−1^ and 1263 cm^−1^ were due to scissoring bending vibrations O–H from hydroxyl groups. FT-IR absorption bands in the region from 1200 to 970 cm^−1^ were mainly due to C–C and C–O stretching in the pyranosyl ring and to C–O–C stretching vibrations of glycosidic bonds [9]. The bands at 1120 cm^−1^ were characteristic of C–O–C ring stretching vibrations from glycoside linkage. The bands at 1028–1029 cm^−1^ were assigned with C–O stretching vibrations, together with bands at 987 cm^−1^. In the fingerprint region was observed typical bands for inulin and inulin-type fructans. The band at 935 cm^−1^ was attributed to the presence of *α*-D-glucopyranosyl residue in the polysaccharide chain. The band at 873 cm^−1^ was due to *β*-anomer bendings in C1–H and 818 cm^−1^ confirmed the presence of 2-ketofuranose or 2-ketopyranose. Similar bands in the FT-IR spectra were reported earlier for inulin isolated by microwave extraction from echinacea roots [10]. The bands at 935, 873, and 818 cm^−1^ were typical for inulin from different plant sources such as Jerusalem artichoke, Globe artichoke [9], chicory [11], burdock [12], dahlia [13], and black salsify [14].

**Figure 2:**
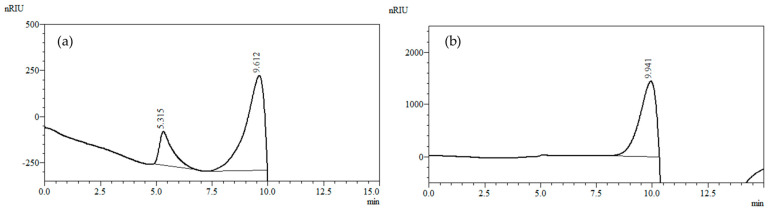
High-performance size-exclusion chromatography (HPSEC) elution pattern of (**a**) PSC_H2O_; (**b**) inulin standard with DP 25 (M_w_ 4050 Da).

The molecular weight distribution pattern of the isolated PSC_H2O_ is shown in Figure 2, while the other chromatograms for the assay of PSCs are arranged in Figure S2a–f. It can be seen that PSC_H2O_ consisted of two polymer/polysaccharide fractions with different molecular weight distributions. The first peak was positioned at 5.0 min and 7.5 min covering the 78.8 kDa and 40 kDa mass range, while the second peak was eluted at a retention time between 7.5 and 10 min. Keeping in mind the retention time (9.941 min) and that of the standard used, it can be ascribed to a relatively low molecular weight compound having <0.59 kDa molecular weight. Bearing in mind the composition of PSCs and the retention time, the first peak can be ascribed to (glyco)protein, pectin, or arabinogalactan associated with protein. The second one, on the other hand, can be ascribed to an inulin fraction with DP 24–25. This inulin can be characterized as a long-chained one. More specifically, it is easy to be observed that after integration of the chromatograms (Figure 2a), the high molecular weight populations (RT 5.0–7.5 min) occupied a smaller percentage (25%) of the total peak area (100%), hence a smaller percentage of PCS_H2O_. As a consequence, the percentage of lower molecular weight fraction occupied a higher percentage (75%), and thus a higher part of the PSC_H2O_. This was consistent with the compositional data (Table 4). As regards the other PSCs, the molecular weight distribution followed a similar pattern (Figure S2). 

## 3. Discussion

In general, the current study examines echinacea roots and investigates the amount of desired phytochemicals that tend to accumulate in the extracts. However, it is known that several factors in the process of extracting the raw material play a crucial role in obtaining a maximum amount of phytoconstituents. One of them is the type and concentration of the solvent, followed by temperature and duration [15].

Our research findings indicated that inulin, cellulose, and uronic acids were the main carbohydrate constituents of coneflower roots. Unlike lipophilic compounds and caffeic acid derivatives, knowledge about the concentration and composition of polysaccharides in the roots, and especially in the extracts, is still very scarce. Concerning inulin, Petkova and Denev investigated the fructan content of *Echinacea purpurea* and found that the content of inulin (12.3%) in the roots was higher [10]. The same authors quantified fructose and glucose in smaller amounts [10]. Bauer noted that other scientists had found an inulin content of 5.9% for the roots of *Echinacea angustifolia* [5]. It may be that the quantitative differences in fructan content are due to the raw material origin, time of harvest, environmental conditions, and seasonal variations. 

Cichoric acid was found as a major constituent in the different parts of the echinacea species. Unlike polysaccharides, there are a large number of papers concerning levels of cichoric acid and other caffeic acid derivatives in the echinacea roots. Interestingly, differences in cichoric acid levels among plant tissue have been reported [16]. It was found that cichoric acid is predominantly present in the aerial parts (flowers) of the plant. Its level in the roots has been quantified to be 0.6–2.1% [5] which was comparable to our findings. A previous study by Wills and Stuart showed that levels of cichoric acid in Australian-grown *E. purpurea* root ranged between 5 and 25 mg/g, which was consistent with our results [17]. Our findings were also in line with the levels of cichoric acid in the roots of German-grown *E. purpurea* (0.76%) [18]. Moreover, it seems that cichoric acid levels strongly depend upon the season and the stage of development of the plant tissue. For example, flowering, mature, and senescent roots contain 30.6, 26.6, and 11.5 mg cichoric acid per g dry wt. [16].

The other main phenolic constituent in *E. purpurea* roots is caftaric acid whose levels seem to vary significantly through the growing season [19]. Our caftaric acid level (Table 1) was lower than that reported by Perry et al. of 0.35–0.41% *w*/*w* in the roots. However, the caftaric acid-to-cichoric acid levels ratio (0.2), used for distinguishing root extracts from extracts of the aerial part, did not differ significantly from that reported by Perry et al. (0.18–0.22) [19]. 

One of the most common forms of echinacea root uses and applications is extracts. Their composition, however, depends on the solvents and technics employed. For example, a study by Babaeva et al. reported that a larger quantity of inulin had been extracted by infusion with 40% ethanol (0.70%) than with 70% ethanol (0.51%) [20]. Our research findings agree with this statement. The most likely explanation is that 70% ethanol precipitated more of the inulins in the plant matrix contrary to 40% ethanol, and thus the concentration of inulin in 70% ethanolic extract was lower [20]. The same pattern was followed by protein and uronic acid constituents present in the extracts. Interestingly, plant material was characterized by a high uronic acid content, but they were present in the extracts in a considerably low amount even in the water extract. Probably, a harsher condition should be employed to obtain deep solubilization of uronic acid-containing constituents, as was demonstrated by an earlier study by Dalby-Brown et al., who employed boiling water (three times for 1 h) to extract pectin-like substances [3]. In addition, different conventional and nonconventional (enzyme, microwave, ultrasound, subcritical water, supercritical CO_2_, etc.) extraction methods can be employed to assist polysaccharide extraction more fully, especially of the uronic acid-containing one. A good example of this is our very recent study on subcritical water extraction, which found that the yields of lemon-balm extracts and polysaccharides increased significantly with an increase in temperature and duration [21]. In fact, a detailed study on how subcritical water affects the yield and composition of echinacea extracts is being undertaken by us. It should be noted parenthetically that the polysaccharide composition of extracts depends not only on extraction conditions but also on the plant parts used. For example, different polysaccharides were isolated from the aerial parts, whereas roots contained inulin-type fractions (6 kDa), glycoproteins, and acidic highly branched arabinogalactan-decorated pectin/protein (70 kDa) [6]. Petkova and Denev reported on the isolation of inulin with an average DP 27 (4.3 kDa) from echinacea roots by microwave-assisted extraction [10]. Cozzolino et al. [22] found inulin from Echinacea roots with 4.5 kDa, while Wack and Blaschek using different water and ethanolic solvent mixtures succeeded in isolating inulin from echinacea roots with DP 33–55 [23]. Our findings indicate that PSC comprised mainly of inulin and glycoprotein/protein isolated from the root extracts by employing different solvents but using the same conditions (1:20 (*w/v*), 60 °C, 1 h, dialyzed).

Considering the extractability of polysaccharide components by different solvents, our findings indicated that water was a better solvent by comparison to 60% glycerol and 60% ethanol. In a previous study, Bergeron and Gafner employed 65% glycerol and 65% ethanol for extracting the polysaccharide/glycoprotein fractions from echinacea roots [24]. They found that hot water gave a higher yield (4.42%), whereas the glycerol (3.87%) and ethanol (3.53%) extracts contained smaller amounts of polysaccharide fractions. In our study, the yields of PSC_60%_ and PSC_60%G_ (Table 4) were very close to those reported by these authors. In addition, we also found that the water extraction of the roots yielded higher amounts of PSCs which was in line with the earlier study [24].

As regards phenolic content, our results showed that water extract consisted of a lower phenolic content (581 mg/L), but ethanolic and glycerolic extracts exhibited a higher concentration (730–850 mg/L). These results differ from those reported by a previous study [25]. The authors reported a lower phenolic content (446 mg/L, 247 mg/L) in two *E. purpurea* extracts (E2, E3). The first (E2) extract had been made from the juices of fresh flowers, leaves, seeds, and roots of *E. purpurea*, without the plant’s stems, while the second one (E3) had been produced from the whole plant (with stems) [25]. Therefore, it seems that phenolic content strongly depends on the plant’s part and the solvent used for the extraction. We have seen other examples of this same case recently. Momchev et al. undertook a study on the effect of extraction conditions on the level of caftaric and cichoric acid in glyceric extracts prepared from *E. purpurea* (aerial parts) [26]. The research findings indicated that the levels of both phenolic acids ranged from 6.6 to 50.3 μg/mL and from 7.5 to 155.3 μg/mL. The maximum amounts have been obtained using 90% (*w*/*w*) glycerol, at 70 °C, ultrasound power at 72 W, and a time of 40 min. In a very recent study, the same team of scientists showed that the amount of these phenolic acids varied between 13.07 µg/mL and 31.55 µg/mL and from 61.11 µg/mL to 103.26 µg/mL depending on the glycerol concentration (50, 70, 90% *w*/*w*), temperature, ultrasonication power, and time [27].

## 4. Materials and Methods

### 4.1. Plant Material

Purple coneflower (*Echinacea purpurea* (L.) Moench.) plants were cultivated by the licensed farmer Fatma Kichukova (Gotse Delchev, Blagoevgrad Province, Latitude: 41.5667, Longitude: 23.7333). Fresh roots were harvested in November 2020, dried in the shade, cut into pieces (0.3–1.5 cm), and kept in paper bags before extraction and analysis.

### 4.2. Proximate Composition Analysis of Plant Material

For the determination of moisture content, the milled sample (~1.3 g) was dried in an automated moisture analyzer (KERN DLB, Balingen, Germany) at 105 °C until constant weight. Ash content was determined as the pulverized sample (0.5 g) was placed in a crucible and ignited in a muffle furnace at 550 °C until there was no change in the mass of the sample. For the estimation of crude lipid content, the ground sample (10.0 g) was packed in a cellulose thimble and subjected to an exhaustive extraction with petroleum ether (500 mL) for 8 h in a Soxhlet extractor. The obtained crude extract was dried under vacuum and its weight was used for the calculation of the lipid content. The crude protein content was evaluated by the micro-Kjeldahl method (N × 6.25). The determination of nitrogen expressed as ammonia content of the digested sample was performed by the acetylacetone–formaldehyde colourimetric method using ammonium sulfate as a standard [28]. The total carbohydrate content of the roots was analyzed by the phenol-sulfuric acid method using glucose for the calibration-curve construction [29]. The sample was solubilized in 72% (*w*/*w*) H_2_SO_4_ (1 h, 30 °C), and after dilution with water to 1 M H_2_SO_4_, hydrolysis was completed in 3 h at 100 °C. The obtained hydrolyzate was used as a sample for analysis. The absorbance was measured at 490 nm.

### 4.3. Uronic Acid, Cellulose, and Total Fructan Content

For the estimation of the uronic acid content of roots and PSCs, an automated [1,1′-biphenyl]-3-ol analysis was performed by a continuous-flow analyzer Skalar San^++^ system (Skalar Analytical BV, Breda, The Netherlands) according to the instructions of the manufacturer and Thibault [30]. Absorption was measured at 530 nm and galacturonic acid (12.5–100.0 μg/mL) was used for a calibration curve construction. Initially, the powdered coneflower root was given a preliminary threefold extraction with 70% (*v*/*v*) aqueous ethanol at 50 °C for 1 h to remove small molecules. The solids were separated by centrifugation (18.187× *g*) before each repetition. Further, the residue was washed twice with acetone at room temperature and vacuum dried. Finally, the sample was hydrolyzed as described above (Section 4.2) and an aliquot of hydrolysate was used as a sample for analysis. 

For the detection of cellulose quantity, the following modification of Updegraff’s method was employed: a sample (50 mg) was heated (30 min, 100 °C) with 3 mL of acetic acid-HNO_3_ reagent (acetic acid:H_2_O:HNO_3_ 8:2:1 *v*/*v*/*v*) in a microtube (5 mL) with a locking clip. After cooling the insoluble residue was recovered by centrifugation and washed with deionized water to neutral pH. The obtained residue was solubilized with 72% (*w*/*w*) H_2_SO_4_ (5 mL). After 1 hour’s standing, the sample was transferred with distilled water into a 100 mL volumetric flask. The concentration of carbohydrates was further determined by the phenol-sulfuric acid method using glucose as a standard (Section 4.2) after an appropriate dilution [31].

The total fructan content of the roots was estimated by a resorcinol-thiourea method. Initially, the powdered root was extracted (1:10 *w*/*v*, 20 min, 45 °C) with water in an ultrasonic bath Siel UST 5.7–150 (Siel, Gabrovo, Bulgaria) (45 kHz, 300 W). The filtered extract (100 μL) was transferred in a 10 mL glass tube, then 100 μL of 1% solution of resorcinol in ethanol (95% *v*/*v*), 100 μL thiourea (0.1% ethanol solution), 800 μL ethanol (95% *v*/*v*), and 900 μL concentrated HCl were added. The samples were placed into a water bath at 80 °C for 8 min. Then the cooling was performed. The glass tubes were filled with water to reach the final volume of 10 mL. The absorbance was measured at 480 nm against distilled water and fructose (0.5–10 mg/mL) was used for a calibration-curve construction [32]. 

### 4.4. High-Performance Liquid-Chromatography Analysis of Inulin and Sugars 

The determination of the quantity of inulin and fructooligosaccharides was carried out using an HPLC instrument Elite Chrome Hitachi with a Shodex^®^ Sugar SP0810 column (300 × 8.0 mm i.d.) with Pb^2+^ ions and a guard column Shodex SP-G (5 μm, 6 × 50 mm) and a refractive index detector Chromaster 5450 (Hitachi High-Technologies Corporation, Tokyo, Japan). The column temperature was 85 °C. The elution of analytes was performed with distilled water at a flow rate of 1.0 mL/min and the volume of injection was 20 μL [33].

### 4.5. High-Performance Liquid-Chromatography Analysis of Caftaric Acid and Cichoric Acid 

The quantitative estimation of caftaric acid and cichoric acid was performed according to the method described in European pharmacopoeia [4]. Briefly, about 0.50 g of the powdered sample and 80 mL of a 70% (*v*/*v*) solution of ethanol were placed in a 100 mL volumetric flask. The mixture was sonicated for 15 min and then diluted to 100 mL with the same solvent. After filtration through a PTFE filter (0.45 µm), the filtrate was used for liquid chromatographic analysis. The separation was carried out on an Agilent TC-C18(2) column (4.6 × 250 mm, 5 µm; Agilent, Santa Clara, CA, USA) joined to a Nexera-*i* LC-2040C Plus UHPLC system (Shimadzu Corporation, Kyoto, Japan) with a UV detector operating at 330 nm. The elution of the sample (10 µL) was conducted at 35 °C and a flow rate of 1.5 mL/min with mobile phase (A)—phosphoric acid:water (1:999 *v*/*v*) and (B)—acetonitrile mixed at the following gradient: 0 min—90% (A); 0–13 min 90%→78% (A); 13–14 min 78%→60% (A); 14–20 min 60% (A). The location of peaks due to different acids was confirmed by relative retention regarding the chlorogenic acid standard: caftaric acid = about 0.8; cichoric acid = about 2.3. The calculation of the percentage content of each phenolic acid in the sample was made using the equations described in the corresponding monograph [4].

### 4.6. Preparation of Extracts

The ground roots were extracted with five different solvents (distilled water, 40%, 50%, 60% (*v*/*v*) ethanol, and 60% (*v*/*v*) glycerol) in separate experiments. The plant material and the corresponding solvent were mixed at a ratio of 1 to 20 (*w*/*v*) and further extracted at 60 °C for 1 h on a shaking water bath. After cooling, the solid was separated from the liquid through a Büchner funnel (filter paper, KA-4, Prague, Czechia). In the case of the 60% glycerol extraction, the separation was performed by centrifugation (3150× *g* for 15 min at 5 °C). The different extracts were designated as E_H2O_, E_40%_, E_50%_, E_60%_, and E_60%G_. The solid residues (without the 60% glycerol residue) were dried at 50 °C to a constant weight. Furthermore, the solid residues of the 40%, 50%, and 60% (*v*/*v*) ethanol-extracted roots were re-extracted with water and 60% (*v*/*v*) glycerol, as described above. The obtained extracts were designated as E_40%/H2O_, E_50%/H2O_, E_60%/H2O_, E_40%/G_, E_50%/G_, and E_60%/G_.

#### Total Phenolic, Flavonoid, and Condensed Tannin Content

The estimation of the quantity of total phenolic, flavonoid, and tannin contents in the initial plant material and the extracts was performed by the method of Singleton and Rossi with Folin–Ciocalteu’s reagent, AlCl_3_ reagent, and methylcellulose precipitation assay, respectively, as described by Ognyanov et al. [34].

### 4.7. Isolation of the Polysaccharide Complexes 

To investigate the polysaccharide complexes that comprised different extracts, an additional amount of extracts were prepared as described in 4.6. Additionally, the volume of the extracts was reduced 3 or 4 times through a lab rotary evaporator. The concentrated extracts were dialyzed extensively (72 h, 4 °C) against distilled water employing MEMBRA-CEL^®^ dialysis tubing (mwco 3.5 kDa; SERVA Electrophoresis), and then freeze-dried.

### 4.8. Physicochemical and Spectroscopic Characterization of Polysaccharides

#### 4.8.1. General Analytical Methods

The protein content was estimated by the dye-binding method of Bradford using Coomassie^®^ Brilliant blue G-250 dye (Amresco^®^) and bovine serum albumin as a standard [35]. The total uronic acid content was estimated by the colourimetric [1,1′-biphenyl]-3-ol method as described above (Section 4.3). The total carbohydrate content was evaluated by the phenol-sulfuric acid method using glucose as a standard as described above (Section 4.2). The total fructose content was estimated by a resorcinol-thiourea method as described above (Section 4.3). For the corresponding analyses, a suitable quantity of the PSC was dissolved in water to obtain a solution having a concentration within the range of the standard curve and run directly by the methods. 

#### 4.8.2. Molecular Weight Distribution Analysis

For the determination of molecular weight distribution pattern, PSC samples were run on a Nexera–*i* LC-2040C Plus UHPLC system (Shimadzu Corporation, Kyoto, Japan) comprised of an Agilent Bio SEC-3 (300 Å, 4.6 × 300 mm, 3 µm) column connected to a refractometric detector 20A. The elution was carried out at 30 °C with a mobile phase of 150 mM NaH_2_PO_4_ (pH 7.0) employing a flow rate of 0.5 mL/min. Pullulan standards (Shodex P-82 kit, Showa Denko, Kawasaki, Japan) with molecular weights in the range of 0.59 × 10^4^ to 78.8 × 10^4^ g/mol and chicory inulin standard Rafliline HPX (DP 25) from Beneo (Orafti, Oreye, Belgium) were used as calibration standards. 

#### 4.8.3. FT-IR Spectroscopy

The FT-IR spectra of the PSCs samples (4 mg) were recorded over a wave number range of 4000–400 cm^−1^ using the attenuated total reflection technique on Tenzor 27 (Bruker, Bremen, Germany) spectrometer, controlled by OPUS 8.7. software. The two spectra were analyzed using SpectraGryph software (version 1.0) (Dr. Friedrich Menges).

### 4.9. Statistical Analysis

All extractions were performed in duplicates. The HPLC analyses were performed at least in duplicates, whereas the other analyses were run at least in triplicates. Results were expressed as mean values ± standard deviations if applicable. One-way analysis of variance (ANOVA) and Student’s *t*-test were used to evaluate the differences in the mean between groups. Any *p* values less than 0.05 were considered to be significant. Microsoft Excel, 2016 (Microsoft Corporation, Redmond, WA, USA) was used in the analyses. 

## 5. Conclusions

In the current study, we quantified different phytochemical constituents of purple coneflower roots. In addition, we characterized different water, ethanolic, and glycerin extracts. It was found that water solubilized better polymer (polysaccharide and protein) constituents, whereas ethanolic solvents extracted better phenolic components. Our study provides many insights into the composition of PSCs and molecular weight distribution. PSCs were represented by long-chained inulin accompanied by different amounts of protein and uronic acid-containing constituents. Cichoric acid was the main phenolic acid constituent in the extracts better solubilized by ethanolic solvents. It was revealed that 60% ethanol and 60% glycerol are equally efficient in extracting cichoric and caftaric acids from echinacea roots. However, from a purely practical point of view, it can be compromised that 40% ethanol and to a lesser extent 50% ethanol can be used for preparing extracts having appreciative amounts of phenolic and polysaccharide/protein constituents. There is a possibility of application after the preparation of extracts with 60% alcohol and subsequent extraction of the residual material with water. As a result, it can be obtained with phenolic acids and simultaneously inulin-rich extracts from one plant source. Our findings can be used as a basis for composing different echinacea products. 

## Figures and Tables

**Table 1:** Chemical characterization of purple coneflower root (*w*/*w*%).

Constituents	Amount %
A. Moisture	12.0 ± 0.1
B. Crude protein (N × 6.25)	20.2 ± 0.3
C. Total lipids	0.8 ± 0.1
D. Total carbohydrates	45.3 ± 0.8
Glucose (Glc)	0.5 ± 0.0
Fructose (Fru)	1.0 ± 0.1
Sucrose (Suc)	0.5 ± 0.0
Total fructans	7.1 ± 1.0
Inulin	6.3 ± 0.7
Nistose	0.3 ± 0.1
1-Kestose	0.3 ± 0.1
Uronic acids	7.7 ± 0.2
Cellulose	14.3 ± 0.6
E. Ash	4.7 ± 0.5
F. Total phenolic content	1.5 ± 0.0
Total flavonoids content	<0.1
Total tannins	<0.1
G. Phenolic acids	0.84
Caftaric acid	0.14 ± 0.01
Cichoric acid	0.7 ± 0.05

Results are presented as mean values ± SD.

**Table 2:** Yield and chemical characterization of purple coneflower root extracts (*w*/*w*%).

Constituents	E_H2O_	E_40%_	E_50%_	E_60%_	E_60%G_
A. Yield	53.1 ± 0.2 ^a^	38.2 ± 0.1 ^b^	33.4 ± 0.2 ^c^	32.3 ± 0.3 ^d^	-
B. Crude protein (N × 6.25)	28.9 ± 0.5 ^a^	26.7 ± 0.3 ^b^	26.9 ± 0.6 ^b^	26.6 ± 0.4 ^b^	-
D. Total carbohydrates	11.2 ± 0.6 ^a^	10.9 ± 0.5 ^a^	9.8 ± 0.2 ^b^	8.8 ± 0.4 ^c^	10.2 ± 0.3 ^b^
Glucose (Glc)	1.4 ± 0.1 ^a^	1.2 ± 0.2 ^ab^	1.3 ± 0.1 ^ab^	1.2 ± 0.1 ^b^	1.0 ± 0.1 ^b^
Fructose (Fru)	3.7 ± 0.2 ^a^	3.2 ± 0.1 ^b^	3.2 ± 0.1 ^b^	3.1 ± 0.3 ^b^	3.0 ± 0.2 ^b^
Sucrose (Suc)	0.3 ± 0.1 ^c^	1.8 ± 0.2 ^a^	1.4 ± 0.2 ^ab^	1.2 ± 0.2 ^b^	1.8 ± 0.2 ^a^
Total fructans	7.0 ± 0.4 ^a^	7.0 ± 0.5 ^a^	6.3 ± 0.2 ^b^	5.3 ± 0.7 ^c^	6.2 ± 0.7 ^ab^
Inulin	7.0 ± 0.2 ^a^	4.6 ± 0.3 ^b^	2.2 ± 0.1 ^c^	0.5 ± 0.1 ^d^	-
Nistose	0.4 ± 0.1 ^b^	1.5 ± 0.2 ^a^	0.2 ± 0.1 ^b^	0.3 ± 0.1 ^b^	0.2 ± 0.1 ^b^
1-Kestose	0.5 ± 0.1 ^b^	1.3 ± 0.1 ^a^	0.3 ± 0.1 ^b^	0.2 ± 0.1 ^bc^	0.1 ± 0.1 ^c^
Uronic acids	0.1 ± 0.0 ^b^	0.2 ± 0.0 ^a^	0.1 ± 0.0 ^b^	0.1 ± 0.0 ^b^	0.3 ± 0.0 ^a^
F. Total phenolic content	1.16 ± 0.02 ^d^	1.53 ± 0.04 ^b^	1.48 ± 0.03 ^c^	1.46 ± 0.02 ^c^	1.70 ± 0.02 ^a^
Total flavonoids content	0.09 ± 0.01 ^c^	0.14 ± 0.01 ^b^	0.14 ± 0.01 ^b^	0.15 ± 0.01 ^ab^	0.16 ± 0.01 ^a^
G. Phenolic acids (total)	0.13	0.69	0.75	0.82	0.76
Caftaric acid	0.06 ± 0.01 ^b^	0.13 ± 0.01 ^a^	0.13 ± 0.01 ^a^	0.13 ± 0.01 ^a^	0.14 ± 0.01 ^a^
Cichoric acid	0.07 ± 0.01 ^d^	0.56 ± 0.01 ^c^	0.62 ± 0.01 ^b^	0.69 ± 0.02 ^a^	0.62 ± 0.01 ^b^

Results are presented as mean values ± SD. There are no significant differences among values marked with the same letters (a, b, c, d) within individual groups (columns of the table).

**Table 3:** Yield and chemical characterization of purple coneflower root extracts of residue after extraction (*w*/*w*%).

Constituents	E_40%/H2O_	E_50%/H2O_	E_60%/H2O_	E_40%/G_	E_50%/G_	E_60%/G_
A. Yield	8.5 ± 0.4 ^c^	11.8 ± 0.3 ^b^	17.3 ± 0.5 ^a^	-	-	-
B. Crude protein (N × 6.25)	34.3 ± 0.3 ^a^	30.3 ± 0.2 ^b^	30.2 ± 0.4 ^b^	-	-	-
C. Total carbohydrates	2.0 ± 0.1 ^c^	2.5 ± 0.2 ^b^	3.8 ± 0.3 ^a^	1.0 ± 0.1 ^d^	1.2 ± 0.1 ^d^	1.8 ± 0.1 ^c^
Glucose (Glc)	<0.1	<0.1	<0.1	-	-	-
Fructose (Fru)	0.1	0.1	0.1	-	-	-
Sucrose (Suc)	<0.1	<0.1	<0.1	-	-	-
Total fructans	1.2 ± 0.2 ^c^	1.5 ± 0.1 ^b^	2.4 ± 0.2 ^a^	0.7 ± 0.1 ^d^	0.8 ± 0.2 ^d^	1.4 ± 0.2 ^b^
Inulin	1.0 ± 0.1 ^c^	2.1 ± 0.3 ^b^	5.0 ± 0.1 ^a^	-	-	-
Nistose	0.0	0.0	0.0	-	-	-
1-Kestose	0.5 ± 0.2 ^a^	0.5 ± 0.1 ^a^	0.4 ± 0.1 ^a^	-	-	-
Uronic acids	0.2 ± 0.0 ^b^	0.2 ± 0.0 ^b^	0.2 ± 0.0 ^b^	0.3 ± 0.0 ^a^	0.3 ± 0.0 ^a^	0.3 ± 0.0 ^a^
D. Total phenolic content	0.43 ± 0.01 ^d^	0.52 ± 0.01 ^b^	0.66 ± 0.02 ^a^	0.38 ± 0.00 ^e^	0.47 ± 0.01 ^c^	0.66 ± 0.02 ^a^
Total flavonoids content	0.043 ± 0.005 ^c^	0.051 ± 0.001 ^b^	0.063 ± 0.002 ^a^	0.026 ± 0.001 ^d^	0.039 ± 0.001 ^e^	0.066 ± 0.002 ^a^

Results are presented as mean values ± SD. There are no significant differences among values marked with the same letters (a, b, c, d, e) within individual groups (columns of the table).

**Table 4:** Yield and chemical characterization of PSCs isolated from purple coneflower root extracts (*w*/*w*%).

PSC	Yield	Protein	Total Carbohydrates	Total Fructans	Uronic Acids
PSC_H2O_	8.87 ± 0.03 ^a^	16 ± 0.3 ^b^	38 ± 0.1 ^f^	35 ± 0.2 ^f^	1.2 ± 0.0 ^e^
PSC_40%_	5.76 ± 0.02 ^c^	12 ± 0.1 ^f^	42 ± 0.2 ^c^	40 ± 0.1 ^c^	1.0 ± 0.0 ^g^
PSC_50%_	5.42 ± 0.01 ^d^	13 ± 0.1 ^e^	36 ± 0.1 ^g^	34 ± 0.1 ^g^	1.0 ± 0.0 ^g^
PSC_60%_	3.58 ± 0.02 ^f^	14 ± 0.1 ^d^	35 ± 0.1 ^i^	34 ± 0.1 ^g^	1.1 ± 0.0 ^f^
PSC_60%G_	2.38 ± 0.01 ^h^	12 ± 0.1 ^f^	40 ± 0.1 ^e^	36 ± 0.1 ^e^	1.1 ± 0.0 ^f^
PSC_40%/H2O_	2.80 ± 0.01 ^g^	14 ± 0.2 ^d^	41 ± 0.2 ^d^	39 ± 0.1 ^d^	4.5 ± 0.1 ^a^
PSC_50%/H2O_	4.70 ± 0.02 ^e^	11 ± 0.1 ^g^	43 ± 0.2 ^b^	42 ± 0.1 ^b^	3.2 ± 0.0 ^c^
PSC_60%/H2O_	6.37 ± 0.03 ^b^	11 ± 0.1 ^g^	45 ± 0.3 ^a^	44 ± 0.2 ^a^	2.8 ± 0.0 ^d^
PSC_40%/G_	0.43 ± 0.01 ^j^	18 ± 0.4 ^a^	25 ± 0.1 ^k^	20 ± 0.0 ^j^	3.7 ± 0.1 ^b^
PSC_50%/G_	0.44 ± 0.01 ^j^	16 ± 0.4 ^b^	28 ± 0.1 ^j^	22 ± 0.1 ^i^	3.5 ± 0.1 ^b^
PSC_60%/G_	0.76 ± 0.01 ^i^	15 ± 0.2 ^c^	35 ± 0.2 ^h^	24 ± 0.0 ^h^	2.8 ± 0.0 ^d^

Results are presented as mean values ± SD. There are no significant differences among values marked with the same letters (a, b, c, d, etc.) within individual groups (rows of the table).

## Data Availability

Not applicable.

## Supplementary Materials

The following supporting information can be downloaded at: https://www.mdpi.com/article/10.3390/molecules28093956/s1, Figure S1: Chromatogram for the assay of caftaric acid and cichoric acid in purple coneflower root; Figure S2: High-performance size-exclusion chromatography (HPSEC) elution pattern of PSCs.

## Sample Availability

Samples of the compounds are available from the authors.
