# Supplementary material for: Phytochemical Characterization of Purple Coneflower Roots (Echinacea purpurea (L.) Moench.) and Their Extracts
Source: Molecules. 2023 May 8;28(9):3956. doi: 10.3390/molecules28093956 (PMC10180171; doi:10.3390/molecules28093956)
Supplement: Supplementary file 1 [file molecules-28-03956-s001.zip › molecules-2320491-supplementary.pdf]

SUPPLEMENTARY MATERIAL

Phytochemical characterization of purple coneflower roots (*Echinacea purpurea* (L.) Moench.) and their extracts

Ani Petrova [1], Manol Ognyanov [1,*], Nadezhda Petkova [2], Petko Denev [1,*]

**Corresponding author. Tel./Fax: +359 32 642759; E-mail: manol.ognyanov@orgchm.bas.bg; M. Ognyanov, Ph.D.; petko.denev@orgchm.bas.bg; P. Denev, Ph.D.*

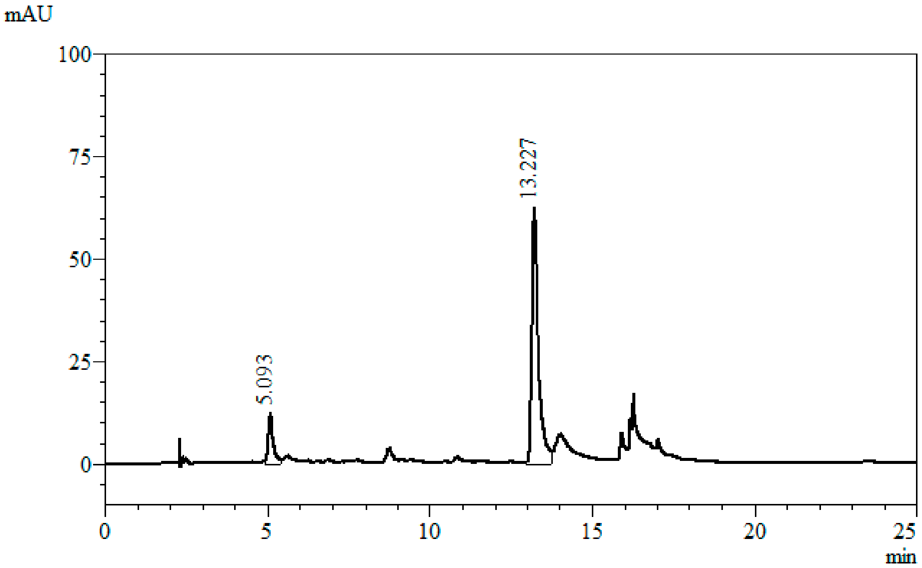

Figure S1. Chromatogram for the assay of caftaric acid (RT – 5.093 min) and cichoric acid (RT – 13.227 min) in purple coneflower root

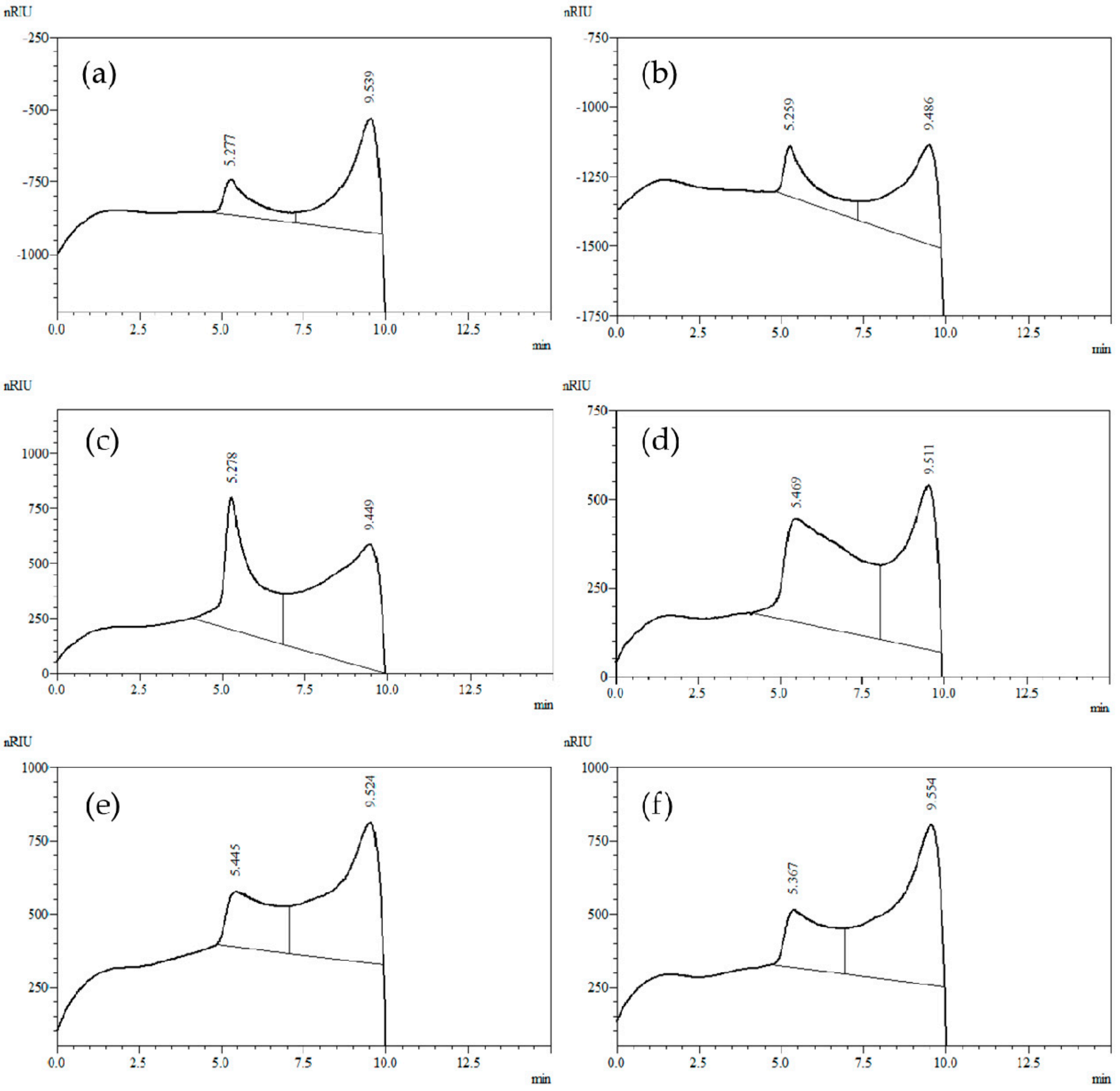

Figure S2. High-performance size-exclusion chromatography (HPSEC) elution pattern of PSCs: (a) PSC$_{40\%}$; (b) PSC$_{50\%}$; (c) PSC$_{60\%}$; (d) PSC$_{40\%/H2O}$; (e) PSC$_{50\%H2O}$; (f) PSC$_{60\%/H2O}$.
